# Pore Structure Influence on Properties of Air-Entrained Concrete

**DOI:** 10.3390/ma18122885

**Published:** 2025-06-18

**Authors:** Kamil Zalegowski

**Affiliations:** Department of Building Materials Engineering, Faculty of Civil Engineering, Warsaw University of Technology, Al. Armii Ludowej 16, 00-637 Warsaw, Poland; kamil.zalegowski@pw.edu.pl

**Keywords:** air-entrained concrete, image analysis, pore structure, microstructure, *UPV*

## Abstract

The study investigates the influence of an air-entraining admixture on the properties and pore structure of ordinary concrete. The aim was to examine how modifications to the concrete mix affect compressive strength, ultrasonic pulse velocity, and resistance to freeze–thaw cycles. Concrete samples with varying admixture dosages (0.00–1.50% of cement mass) were tested for mechanical properties and pore structure. Freeze–thaw resistance was assessed using both direct (PN-B-06265) and indirect methods (EN 480-11), while pore characteristics were evaluated via computer-aided image analysis. Results show that increasing the admixture dosage enhances freeze–thaw resistance by refining the pore structure—particularly by increasing the content of micropores below 0.3 mm—while simultaneously reducing compressive strength and ultrasonic velocity. Statistical analysis revealed that pore parameters such as total air content, specific surface area, and spacing factor significantly correlate with concrete performance. The regression models confirmed that compressive strength and ultrasonic velocity are negatively impacted by increased pore volume, while freeze–thaw resistance improves due to a more favorable pore size distribution. The findings demonstrate that optimizing the admixture dosage can effectively balance durability and mechanical performance, and that quantitative stereological parameters provide a valuable basis for predicting the behavior of air-entrained concrete.

## 1. Introduction

The behavior of every material is somehow related to its microstructure. It is well known that changes in pore content, their size distribution, and even in their arrangement in hardened cement matrix may have a significant effect on the performance of cement composites [1,2,3,4,5,6,7,8,9]. A clear example of this is how the pore structure affects concrete durability in cold climates. The durability of concrete is one of the key factors determining the quality and longevity of concrete structures. A particularly significant factor influencing concrete durability and safe use of structures in such conditions is resistance to the aggressive impact of the environment caused by cyclic freezing and thawing.

In hardened concrete, there are three main types of pores: gel pores, capillary pores, and air voids [10,11,12,13]. Gel pores are very small (less than 10 nm) and do not affect the strength or durability of concrete, as they are inaccessible and do not participate in water transport. Capillary pores, which can be up to 1 μm in size, do not influence the compressive strength, but they are very important for durability (including freeze–thaw resistance), because they form a system of pathways for water and aggressive ion transport. When the temperature drops well below freezing, water inside these pores starts to freeze. As water turns into ice, it expands by about 9% [14,15,16]. This volume expansion leads to pressure buildup within the pores if the matrix lacks sufficient pore space and interconnectivity to accommodate it [14]. If the pressure surpasses the tensile strength of the cement paste at any point, local cracks will form, and consequently, the mechanical properties and durability of the concrete may deteriorate after a number of freeze–thaw cycles [16,17,18,19,20]. Certainly, the intensity of the induced hydraulic pressure depends on the cement paste’s permeability, degree of saturation, distance to the nearest unfilled void, and the freezing rate [21].

The third type of pore that can be distinguished in the concrete microstructure are air voids, also called entrapped air. Their content in ordinary concrete is about 3% of the volume, and their size can be as large as 5 mm [22]. Air voids are irregular in shape, mostly formed accidentally during mixing, transport, and other handling properties. They do not contribute to improved freeze–thaw resistance; on the contrary, they may negatively affect durability and significantly deteriorate mechanical performance [23,24,25,26].

Concrete can be made more resistant to freezing and thawing by changing its microstructure using an air-entraining admixture (*AEA*) [27,28,29]. This admixture lowers the surface tension of the mixing water, facilitating the formation and stabilization of fine air bubbles within the cement paste. High alkalinity in the mix further makes this effect even stronger, promoting the generation and persistence of these tiny air voids. They are smaller than the entrapped air, have a spherical shape and are usually between 0.01 mm and 0.1 mm [30] or, in some cases, up to 0.3 mm [31]. These introduced microscopic, uniformly distributed air voids interrupt the continuity of capillaries in the concrete and provide a pressure relief system. When water within the concrete freezes and expands, the nearby air voids gives the ice space to grow, which reduces internal stress and prevents cracking [32]. Besides improving freeze–thaw resistance, air-entraining admixture may also improve the concrete fire resistance [33], reduce water absorption (decrease in the penetration depth and absorbed water) [34,35,36], as well as workability of fresh mix [37,38].

Although the *AEA* have many benefits, the resulting increase in air content may cause some properties concerns, especially when the dosage is too high or due to unfavorable technological conditions. In general, higher air content is associated with a reduction in the compressive strength of concrete—for every 1% increase in air content, the compressive strength can drop by 4% to 6% [39,40]. The introduced micropores may lead to increase in apparent gas diffusivity and permeability [41,42]. Excessive air entrainment may reverse the intended effects of the admixture, leading to reduced workability [43], increased water absorption [44], higher permeability and delayed setting [45], end even aggravate freeze–thaw damage [28,46], due to microstructure deterioration.

As shown, the pore structure of cement composites significantly affects their performance under cyclic freezing and thawing conditions. Therefore, the traditional design process for cement composites—that is based on the relation between composition, production technology and properties—should be supplemented with the analysis of the material’s microstructure. This should include recognizing the quantitative relations between microstructure and properties.

There are many methods of analyzing microstructure of concrete. These methods may be divided into two groups: microscopic techniques, which allow for the observation of microstructure morphology (i.e., dimensions or arrangement of phases at different levels of magnification), and volumetric technics, which provide averaged information about the microstructure within a given sample volume (e.g., mercury porosimetry, nitrogen absorption, image analysis). Especially interesting is the image analysis method, due to its low cost and the short time required to obtain results. In the area of cement composite technology, the image analysis method is commonly employed to calculate parameters that describe the geometrical features of aggregate grains and pore structure, e.g., amount, size, volume fraction, surface, shape, and arrangement [41,47,48,49,50,51,52,53,54,55,56,57,58,59,60]. It can also be used to assess crack characteristics in the hardened cement matrix by determining their length, orientation, and width [61,62,63,64,65,66]. Less common uses include analysis of the degree of cement hydration [67] and the petrographic analysis of concrete [68]. Recently, advanced computer tools like neural networks are being used more often to automatically analyze complex microstructure images, which is shown by the growing number of scientific papers on this topic.

Image analysis can be used to estimate freeze–thaw resistance indirectly by determining pore structure parameters using the transverse method, as described in EN 480-11 [69]. This method has several advantages, the most important one being that it takes much less time than direct testing methods. The procedure involves preparing precisely ground and polished samples and observing the pore structure along a set of parallel measurement lines using an optical or digital microscope. During this, the number of pores intersected by the lines and the length of chord of each pore are recorded. The data is then analyzed to describe the pore system using parameters like total pore content (*A*), content of the micropores below 0.3 mm (*A_300_*), specific surface area (α), spacing factor ($\bar{L}$), and pore size distribution. Although the standard does not define fixed threshold values for these parameters, recommended ranges are available in the literature and national specifications [14,69,70,71,72,73,74,75,76,77,78]. The most important microstructure parameters—mainly because they are the most commonly evaluated and compared in studies—are the $\bar{L}$ and *A_300_*. Typically, $\bar{L}$ should not exceed 0.25 mm, while *A_300_* should be at least 1.5%. The recommended values for the remaining parameters are as follows: *A* between 4% and 7%, *A_300_* at least 1.5%, and α between 15 and 24 mm^−1^.

In this study, image analysis was used to examine the pore structure of ordinary concrete, where the microstructure was modified by adding different amounts of air-entraining admixture. The analysis included parameters listed in EN 480-11, as well as additional ones proposed by the author, such as the share of pores in different size ranges and the pore content excluding coarse aggregate. The tests were carried out using a proprietary computer program developed in MATLAB (ver. R2024b).

Manual analysis using the traditional transverse method is time-consuming, requires experience, and needs a microscope, either optical or digital. The method proposed in this study follows the EN 480-11 sample preparation procedure, but instead of using a microscope, the microstructure image is captured digitally. Then, a two-step image analysis is performed: first, manual segmentation to isolate pores, and second, automatic measurement and calculation of pore structure parameters. This approach makes the analysis faster and more accurate, because the computer performs calculations quickly and consistently. Also, a microscope is not needed, since a high-resolution flatbed scanner can be used to capture the image [79,80,81,82]. Similar methods have been successfully used in other studies [76,77], where computer algorithms were applied to evaluate the air void system in concrete according to EN 480-11. However, unlike those studies, this work compares indirect image-based results with direct freeze–thaw resistance tests.

The European standards do not specify the methodology for freeze–thaw resistance testing or the criteria for its assessment. Therefore, the direct tests were performed according to procedure described in PN-B06265 standard [83], which is the Polish supplement to EN 206 [84]. The applied method enables the assessment of frost resistance under cyclic freezing and thawing conditions, considering both the degree of internal damage to the concrete, expressed by the reduction in sample compressive strength, and external damage, defined by level of surface damage and loss of the sample mass. The results were analyzed using correlation and multiple regression with stepwise selection, to determine which microstructure parameters have the strongest effect on freeze–thaw resistance, and to identify those that can be used to build a predictive model. This study offers a new insight, as such a comparison between direct and indirect methods has not been made before—likely due to the time and effort required for direct freeze–thaw testing.

In designing air-entrained concrete, it is important to find a balance between compressive strength and durability in freeze–thaw conditions. Adding air to concrete helps it resist freezing and thawing, but it also creates tiny air voids. These increase the concrete’s porosity, which lowers its density and reduces its compressive strength. Too much air can make the structure too weak, while too little air increases the risk of damage from freezing and de-icing salts. That is why both the working conditions and structural requirements must be considered to find the right amount of air. The goal is to achieve a good balance between durability and strength. For this reason, a similar analysis was performed for compressive strength as was done for freeze–thaw resistance. The analysis also included the ultrasonic pulse velocity (*UPV*), which is one of the most important non-destructive testing methods. The wave velocity depends on the material’s density, elasticity, and the presence of pores or small cracks. This makes it useful for estimating compressive strength [53,79,85,86,87,88]. Although this method does not directly measure air content, more air voids—common in air-entrained concrete—decreases [89,90]. So, wave velocity can indirectly show how porous the concrete is. Because of this, ultrasound can help assess the concrete’s strength, uniformity, and durability.

The presented article brings new value by applying advanced imaging and statistical methods to a practical problem of designing durable concrete for cold climates. By combining microstructure analysis with mechanical and durability properties, it offers a holistic approach to concrete design that considers both structural and environmental factors. The developed models allow for precise design of freeze–thaw resistant concrete by optimizing the amount of air-entraining admixture. These models can also support quality control in technology labs and may even help update standards for freeze–thaw-resistant concrete design. Finally, they may be used to optimize the dosage of air-entraining admixtures.

## 2. Experimental Investigation

### 2.1. Materials

**Figure 1:**
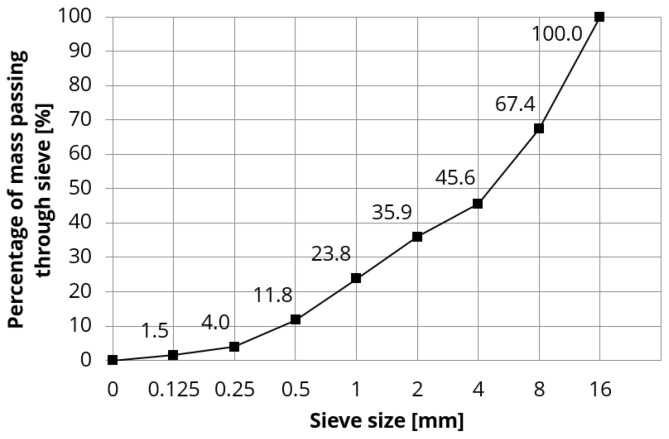
The granulation curve of aggregate used in the study.

In this study, ordinary concrete was modified with an air-entraining admixture. The reference concrete was the ordinary concrete made with Portland cement CEM I 42.5R and pebble aggregate. The aggregate mix included fine aggregate (fraction 0/2 mm), which was river sand, and coarse gravel aggregates with the maximum grain size of 16 mm (fraction 2/4 mm, 4/8 mm, and 8/16 mm)—see Figure 1. The composition of reference concrete was deliberately designed in such a way to produce hardened concrete susceptible to freeze and thaw damage. The water-to-cement ratio of the concrete mixes was fixed at 0.55. According to the EN 206 European standard, concrete with such a w/c ratio would not meet the requirements for freeze–thaw exposure classes XF3 and XF4, i.e., concretes highly saturated with water, with or without anti-icing agents.

The reference concrete was then modified by adding an air-entraining admixture (Table 1), designed to introduce a stable system of microscopic air voids. These voids disrupt the continuity of capillary pores, thereby improving freeze–thaw resistance of cement composites, even in the presence of de-icing agents. The admixture [91] also reduces segregation and bleeding, and it is compatible with all types of cement and other chemical admixtures, including superplasticizers. It is recommended for use in concrete exposed to harsh environmental conditions, such as road pavements, precast elements, and bridge components. The admixture was incorporated into concrete mix at a dosage of 0.04%, 0.2%, and 1.5% of the cement mass, representing the lower limit, recommended dosage, and upper limit suggested by the manufacturer, respectively. A detailed composition of the concretes used in the study is given in Table 2.

### 2.2. Methods

#### 2.2.1. Consistency

The consistency of the concrete mix was tested using the slump test method, according to EN 12350-2 [92]. The fresh concrete was placed into a cone-shaped mold in three layers, with each layer compacted manually by 25 strokes using a tamping rod. After filling, the mold was carefully lifted, and the slump was measured. The test consists of measuring the slump, defined as the difference between the height of the mold and the height of the highest point of the demolded concrete mix sample. The slump test is sensitive to changes in the consistency of the concrete mix and can measure slumps ranging from 10 mm to 210 mm. The entire procedure—from the start of filling the mold to removing it—should be completed within 2.5 min. The consistency classes based on the slump test are shown in Table 3.

#### 2.2.2. Air Content

The air content in the concrete mix was measured using the pressure method according to EN 12350-7 [93], which involves a device called a pressure air meter. It is widely used because it is quick, reliable, and suitable for most types of concrete, especially those containing normal weight aggregates. In this method, a sample of fresh concrete is placed into a sealed container and covered with a lid equipped with a pressure gauge. Water is added to fill the space above the concrete, and then air pressure is applied. When the pressure is released into the chamber, the change in pressure is used to calculate the volume of air in the concrete. The result, shown on the gauge, indicates the percentage of air content with an accuracy of 0.1%. This method is based on the relation between air volume and applied pressure, described by Boyle’s Law, which states that for a given mass of an ideal gas at constant temperature, the volume is inversely proportional to the pressure.

#### 2.2.3. Compressive Strength

The compressive strength tests were carried out on cubic samples measuring 100 mm × 100 mm× 100 mm. The samples were kept in water at a temperature of 20 ± 2 °C for 28 days, following the EN 12390-2 standard [94]. The compressive strength for each concrete mix was calculated as the average of the results from three samples.

#### 2.2.4. Ultrasonic Pulse Velocity

One of the oldest and simplest non-destructive method (*NDT*) is the ultrasonic pulse velocity (*UPV*) testing. The *UPV* method utilizes longitudinal elastic waves—vibrations with the frequencies above 20 kHz, which is the upper limit of the audible frequency for humans. The higher frequency vibrations allow for more accurate measurements; however, they also are subjected to greater attenuation, resulting in lower signal energy. Consequently, the testing of heterogenous concrete composites should be carried out using ultrasonics with the highest possible frequencies that still allow for the registration of good quality waveforms after signal propagation through the material.

**Figure 2:**
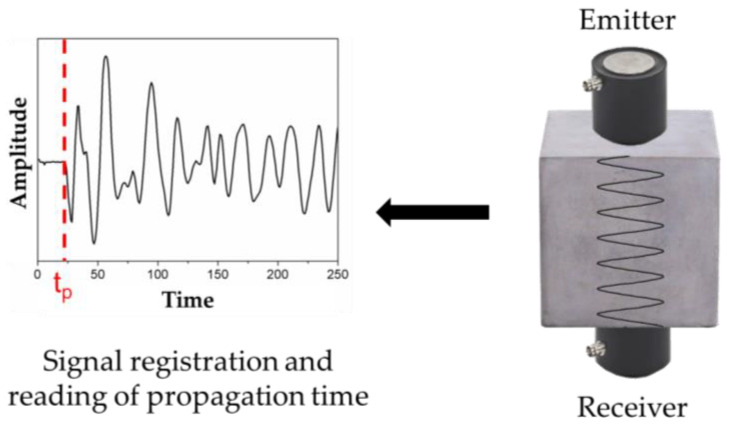
Scheme of ultrasonic measurements.

In this study, a digital ultrasonic flaw detector Pundit PL-200 and two piezoelectric transducers operating at the frequency of 54 kHz were used. The test was performed by the direct method (Figure 2) according to EN 12504-4 [95], where transducers were placed on opposite sides of 100 mm × 100 mm × 100 mm cubic samples, intended for compressive strength tests. To determine the propagation time of the ultrasonic pulse between the emitter and receiver, amplitude-versus-time graphs were analyzed (Figure 2). The *UPV* was calculated by dividing the distance between transducers (equal about 100 mm) by the recorded propagation time. Each measurement was repeated in three different directions per sample. With three samples prepared for each material, a total of nine *UPV* results were obtained for each composition. Finally, four average velocities were calculated for each type of concretes.

#### 2.2.5. Freeze–Thaw Resistance (Direct Method)

The EN-206 European standard [84] does not specify the methodology for freeze–thaw resistance testing or the criteria for its assessment. Therefore, the direct tests were performed according to the procedure described in the PN-B06265 standard [83], which is the Polish supplement to EN 206. The applied method enables the assessment of frost resistance under cyclic freezing and thawing conditions, considering both the degree of internal damage to the concrete, expressed by reduction in sample compressive strength, and external damage, defined by level of surface damage and loss of the sample mass. For the test, 12 cubic samples with dimensions of 100 mm × 100 mm × 100 mm were prepared per each composition and cured in water at a temperature of 20 ± 2 °C for 28 days. After this period, half of the samples were removed from the water, weighed, and subjected to cycles of freezing in air and thawing in water, while the remaining half was left in water as control samples. Each cycle lasted 8 h, with the temperature ranging from −18 ± 2 °C to 18 ± 2 °C. The testing lasted for 150 cycles, corresponding to a total duration of 50 days. Subsequently, the compressive strength of the samples subjected to cycles, as well as reference ones, was assessed. Once all results were collected, the average mass loss (Equation (1)) and the average compressive strength reduction (Equation (2)) were calculated using the following formulas:

(1) $$∆m=\frac{m_{1}-m_{2}}{m_{1}}\cdot 100[\%]$$

(2) $$∆f_{c}=\frac{f_{c1}-f_{c2}}{f_{c1}}\cdot 100[\%]$$

where *m*_1_—the average mass of the samples before the first cycle in a water-saturated state [kg]; *m*_2_—the average mass of the samples after completing the cycles in a water-saturated state [kg]; *f*_*c*1_—the average compressive strength of the control samples (non-frozen, water-saturated) [MPa]; and *f*_*c*2_—the average compressive strength of the samples after the final cycle [MPa].

The freeze–thaw resistance test gives a positive result, i.e., a result confirming the achievement of the freeze–thaw resistance level corresponding to the specified number of cycles (in this case, F150), when the following conditions are met:

- the samples do not show any cracking,
- the average mass loss of the samples does not exceed 5%,
- the average reduction in compressive strength does not exceed 20%.

#### 2.2.6. Pore Structure Analysis (Indirect Method)

The quantitative analysis of pore structure was carried out using image analysis method. It is a stereological method used for quantitative characterization of three-dimensional objects in a material volume based on measurements performed on the two-dimensional images of the microstructure. The analysis is performed on the surface of specially prepared cross-sections of samples, known as microsections. Obtaining accurate and representative results requires precise sampling and proper preparation of the surfaces to remove deformed and damaged layers of material (e.g., those caused during sample cutting). This is achieved through grinding and polishing, which ensure that the microstructure images truly reflect the material. Sample preparation often begins with embedding the specimens in a colored polymer resin. This step protects fragile materials or coatings, standardizes sample sizes to fit into holders for grinding and polishing machines, and before all, allows for distinguishing pores from other microstructural components.

Microstructure images are commonly captured using light microscopes [50,55,59,62] or digital cameras [54,60,64,65]. However, flatbed scanners could also be employed [35,36,37]. These techniques are effective for evaluating microstructures at the macro- or microscale. For finer scale analysis, more advanced tools such as a scanning electron microscope (SEM) [48,56] or X-ray micro-computed tomography [41] are required. The quality of the images, particularly their resolution, determines the smallest pore diameter that can be detected using image analysis.

**Figure 3:**
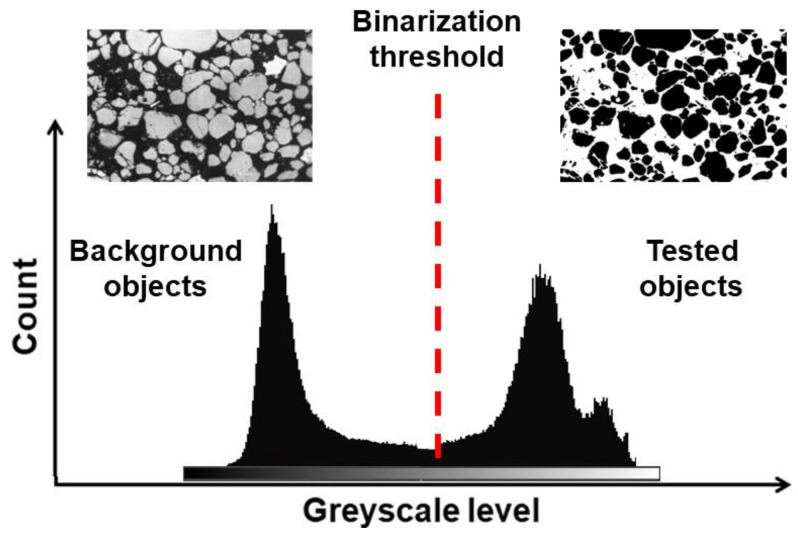
The scheme of image binarization with a global threshold; on the left—output image, on the right—image after segmentation.

The captured images undergo qualitative analysis through digital image processing to differentiate components of the microstructure and isolate those relevant to the analysis. Digital image processing includes any changes to the image’s RGB values—such as adjusting brightness, contrast, or binarization—as well as structural modifications in binary images, like morphological transformations (e.g., dilation or erosion). In addition, the process of isolating specific areas of the microstructure is called segmentation. It is based on the chosen uniformity criterion, such as color, brightness level, or texture. The simplest form of image segmentation is binarization, which separates all image pixels into black (representing the selected microstructural components) and white (representing the insignificant background), based on a specified brightness threshold (Figure 3). Following segmentation, the images undergo quantitative analysis, primarily using computer software, which ensures faster, more accurate, and more consistent results than manual methods.

**Figure 4:**
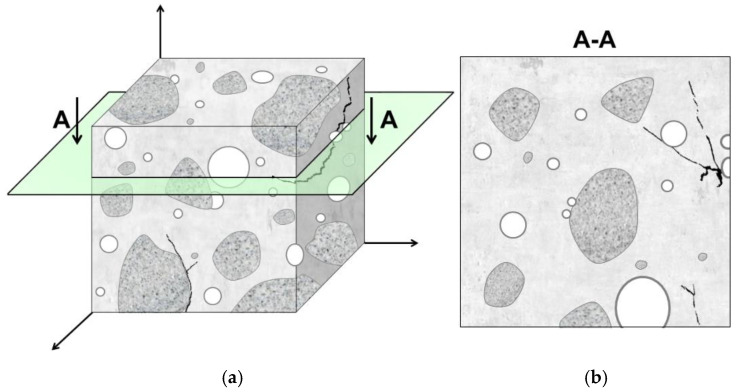
Hypothetical microstructure of concrete containing aggregate grains, pores, and cracks: (**a**) three-dimensional sample with marked cross-section A–A, (**b**) two-dimensional sample cross-section [79].

Capturing a microstructure image reduces the number of dimensions of its constituents (Figure 4). For instance, three-dimensional objects, such as aggregate grains or pores with a volume *V*, are represented as planar shapes with area *A*; the interfacial transition zones between aggregate grains and the hardened cement matrix are depicted as curvilinear lines with a length *L*, while cracks may be linked to points *P*. As a result, parameters describing the three-dimensional microstructure of a material (Table 4, parameters with index *V*) are derived from the characteristics measured on the individual cross-sections (Table 4, parameters with index *A*). One of the fundamental and simplest stereological principles is the Cavalieri–Hacquert relation [96]. It states that the volume fraction of the selected phase within a unit volume of material (*V_V_*) is equal to surface area fraction in the microstructure image (*A_A_*), and also equal to the fraction of grid points hitting that phase in the image (*P_P_*). This relation is expressed as follows (Equation (3)):

*V_V_ = A_A_ = P_P_* (3)

Another example is the relation estimated using the Saltykov method [97]. In this approach a microstructure image is intersected with multiple vertical test lines with a total length *L*. The number of intersections *n* between these lines and the selected objects in the image is counted. Based on this, the intersection density can be calculated using the following equation (Equation (4)):

(4) $$S_{V}=2\frac{n}{L}$$

The image analysis method could also be used to indirectly estimate the freeze–thaw resistance by determining parameters of pore structure according to EN 480-11 [69]. Its main advantage is the significantly shorter testing time compared to direct methods. The procedure involves preparing precisely ground and polished samples, and conducting pore structure observations along a series of parallel measurement lines. During this process, the number of pores intersected by the lines and the chord length of each pore are recorded. The mathematical analysis allows for describing the pore system with parameters such as total pore content (*A*), content of the micropores below 0.3 mm (*A_300_*), specific surface area (α), and spacing factor ($\bar{L}$).

The total air content (*A*) represents the percentage of the total volume of pores within the concrete volume, and it corresponds to the *V_V_*. It is determined as the ratio of the total length of chords passing through pores (*T_a_*) to the total length of the measurement lines (*T_tot_)*, expressed as a percentage (Equation (5)):

(5) $$A=\frac{T_{a}\times 100}{T_{tot}}\;[\%]$$

Pore content with the diameter of up to 0.3 mm (*A_300_*) refers to the percentage of pores with the maximal diameter of 0.3 mm in the total volume of a material. It is determined during the calculation of the air pore size distribution.

The specific surface area of pores (*α*) is a parameter defined as the ratio of the total surface area of the pores to their volume. It is calculated using the following equation (Equation (6)):

(6) $$\alpha =\frac{4\times N}{T_{a}}\;[{mm}^{-1}]$$

where *N* is the total number of chords passing through the pores, and *T_a_* is the total length of the chords passing through the pores.

Pore spacing ratio $\bar{L}$ represents the maximum distance separating any point in the cement matrix and the edge of the nearest pore. It is calculated using one of the two equations, depending on the ratio *R*, which is defined as the ratio of the cement paste volume (computed on the base of the mix recipe) to the total air content. If *R* > 4.342, Equation (7) is used, whereas for *R* ≤ 4.342, Equation (8) must be applied [69]. The aforementioned equations are as follows:

(7) $$\bar{L}=\frac{3\times \left[1.4\times {\left(1+R\right)}^{1/3}-1\right]}{\alpha }\;[mm]$$

(8) $$\bar{L}=\frac{P\times T_{tot}}{400\times N}\;[mm]$$

where *R*—the ratio of the cement matrix volume to the total air content, α—the specific surface area of the pores, *P*—the volume fraction of the cement paste, *T_tot_*—the total length of measurement lines, and *N*—the total number of chords passing through the pores.

**Figure 5:**
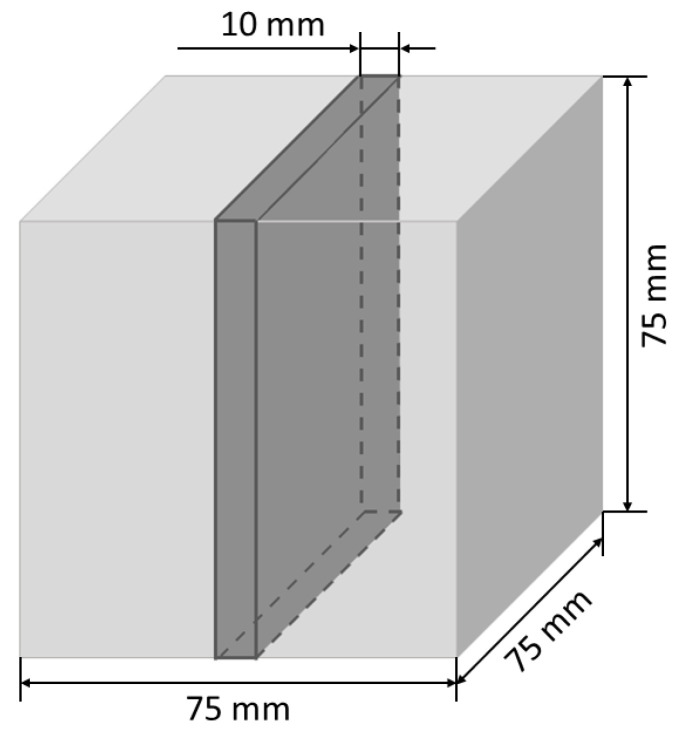
Scheme of sample cutting for computer image analysis.

In this study, the pore structure was analyzed using the method and parameters defined in the EN 480-11 European standard. However, the procedure for determining these parameters was modified. Unlike the transverse method recommended in the standard, the study utilized the image analysis method supported by a proprietary computer software operating in the MATLAB environment (ver. R2024b). For each concrete mix, three samples with dimensions of 75 mm × 75 mm × 75 mm were prepared and cured in water at the temperature of 20 ± 2 °C for 28 days. The preparation procedure involved the following steps: (1) cutting one slice approximately 75 mm × 75 mm × 10 mm in size from each of the three samples for every concrete composition (resulting in a total of three slices per mix composition, as shown in Figure 5); (2) cold mounting the cut slices under reduced pressure using a blue-colored epoxy resin; (3) grinding; (4) final polishing.

**Figure 6:**
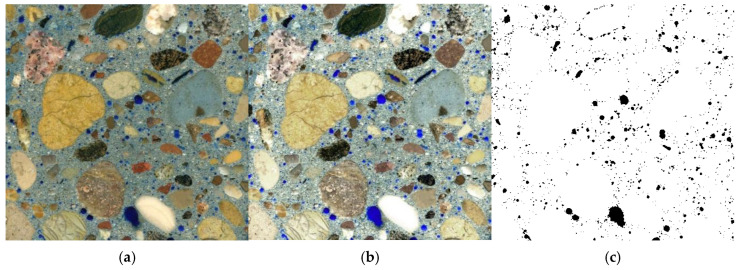
Scheme of image preparation for computer image analysis: (**a**) recorded image; (**b**) processed image; (**c**) binarized image of pore structure.

After preparing the sample cross-sections, 2D images of the microstructure were captured using a flatbed scanner at a resolution of 4800 DPI, where 1 pixel corresponds to 5.3 μm. The resolution of the recorded images was selected to be as high as possible in order to enable the most accurate observation of the smallest pores, without significantly increasing the time required for image processing and analysis. The images were processed using specialized software to obtain precise binary images, isolating the black pores of interest against a white background representing other irrelevant components of microstructure in GIMP—GNU Image Manipulation Program ver. 2.10. The digital image processing involved adjustments of contrast, brightness, gamma, and color saturation (Figure 6b), followed by pore selection and binarization (Figure 6c). As a result of the computer analysis, parameters describing the pore structure were obtained, including those required for assessing freeze–thaw resistance according to EN 480-11; these parameters included the total air content (*A*), content of micropores up to 0.3 mm (*A_300_*), specific surface area of pores (α), and pore spacing ratio ($\bar{L}$). To better understand how the pore structure affects concrete properties, the analysis also included the share of pores with a diameter between 0.3 mm and 1 mm (*A_1000_*) pores equal to or greater than 1.0 mm (*A_MAX_*), and the share of pores excluding coarse aggregate, which represents the share of pores in the cement mortar (*A_C_*)—see Table 5.

#### 2.2.7. Statistical Analysis

In this study the obtained results were subjected to statistical analysis to determine the effect of the air-entraining admixture on the concrete properties and microstructure parameters, as well as to establish relations between them. The relation between variables can be described using correlation and regression [98], both of which were applied in this study. Correlation measures the strength of the relation between two variables, while regression aims to describe this relation using a mathematical model that allows prediction of one variable based on the value of the other. In practice, a common way to check for correlation between two features is to create a scatter plot, where the independent (explanatory) variable is placed on the *x*-axis and the dependent (response) variable is placed on the *y*-axis. The pattern of points on the plot gives an initial idea of the strength and direction of the relationship and can help in choosing a suitable mathematical function to describe it (e.g., linear, polynomial, or exponential).

There are several numerical indicators that express the strength of the relation between two variables. The most commonly used are the Pearson correlation coefficient (*r*) and the coefficient of determination (*r^2^*). The Pearson coefficient is a standardized measure of the strength and direction of a linear relationship, ranging from −1 to 1. A positive or negative sign indicates the direction of the correlation, and the closer the absolute value of *r* is to 1, the stronger is the correlation. The coefficient of determination (*r^2^*) is calculated by squaring the correlation coefficient. It shows what percentage of the variation in the dependent variable can be explained by changes in the independent variable.

Regression analysis is a statistical tool used to examine the relation between quantitative variables and to predict unknown values of a dependent variable based on known values of one or more independent variables (predictors). The regression model equation is usually determined using the least squares method, which fits a line in such a way that the sum of the squared differences between the observed (empirical) and predicted (theoretical) values is minimized. The simplest case is simple linear regression, where the goal is to determine how changes in the dependent variable *Y* can be explained by changes in a single independent variable *x* (Equation (9)):

*Y = β_0_ + β_1_x* (9)

where *β_0_*—constant term, *β_1_*—slope coefficient of regression line.

When the effect of two or more independent variables on variable *Y* is considered, the regression is called multiple regression, and the equation of the line takes the following form (Equation (10)):

*Y = β_0_ + β_1_x_1_ + β_2_x_2_ + … + β_n_x_n_* (10)

The constant term (*β_0_*) is the point where the regression line crosses the *y*-axis. The coefficients *β*_1_, *β*_2_, … *β_n_* are the structural parameters of the model, representing the independent contribution of each predictor (independent variable) to the prediction of the dependent variable. The sign of each coefficient indicates whether the effect of the corresponding independent variable on the dependent variable is positive or negative.

The quality of the regression model is most often evaluated using the multiple correlation coefficient (*R*) and the coefficient of determination (*R^2^*). The *R* value shows the overall strength of the relation between the dependent variable *Y* and all the independent variables included in the model (*x_1_*, *x_2_*, ..., *x_n_*). When squared, *R^2^* becomes a measure that can be interpreted as the percentage of the variation in the dependent variable explained by the model. It is also useful for comparing the quality of different models. However, since *R^2^* increases with the number of predictors, models with different numbers of independent variables are compared using the adjusted *R^2^*, which accounts for model complexity. These regression coefficients can range from 0 to 1, where values closer to 1 indicate a better fit. Another commonly used measure of how well the model fits the data is the standard estimation error (*SEE*), which is the standard deviation of the residuals. It is expressed in the same units as the dependent variable and shows the average difference between the observed values of *Y* and the values predicted by the model.

The statistical analysis of the results presented in this study was conducted using the Statistica data analysis software. Relationships between variables were illustrated using scatter plots with linear trend lines fitted by the least squares method. The strength of these relationships was assessed using Pearson’s correlation coefficient (*r*). Simple linear regression models were also presented.

In the regression analysis, models were developed to best explain the variability of the dependent variables—compressive strength, ultrasonic pulse velocity, and freeze–thaw resistance—based on quantitative parameters describing the pore structure of the tested concretes. The strength of the relations between the dependent variables and the selected set of independent variables (included in the regression models), the significance of the effect of the selected independent variables (pore structure parameters) on the predictive value of the models, and the level of differences between actual observations and values predicted by the models were assessed using the multiple correlation coefficients (*R*), test probabilities (*p*), and standard estimation error (*SEE*), respectively. The usefulness of individual microstructural parameters in explaining the dependent variables was assessed based on the probability values (*p*), while the direction and magnitude of the changes they caused were determined using the *β* regression coefficients.

#### 2.2.8. Summary of Experimental Plan

**Figure 7:**
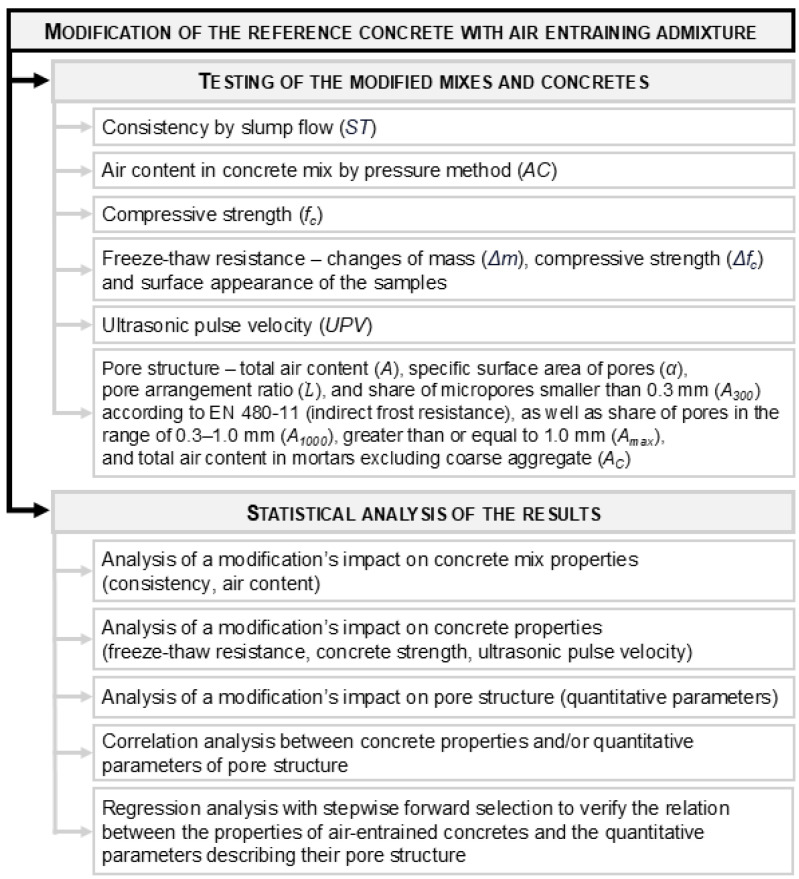
The scheme of the experimental program.

The experimental program is outlined in Figure 7, while Table 6 provides a summary of the tests performed and the number of samples used for each concrete composition.

## 3. Results and Discussion

### 3.1. The Influence of the Air-Entraining Admixture on the Properties and Microstructure

#### 3.1.1. Fresh Mix Properties

The results of concrete mix tests including the consistency by the slump test (*ST*) and the air content (*AC*) by the pressure method are presented in Table 7.

The increase in the amount of air-entraining admixture led to a greater air content. A significant increase in the air content was observed at the admixture level of at least 0.2% (more than 2 times compared with *N0*). At the highest tested dose (1.5%), the *AC* was over 3.5 times higher than *N0*, showing that the admixture became less effective at higher doses. Importantly, just 0.2% was sufficient to meet the air content requirement specified in the EN 206 standard (minimum air content of 4%), necessary to ensure adequate durability of concrete in freezing–thawing conditions (exposure classes XF2–XF4). The effectiveness of the admixture decreased after exceeding the amount recommended by the manufacturer (i.e., 0.2%). This means that adding more admixture does not cause a proportional increase in entrained air.

The admixture had a strong influence on consistency, increasing the fluidity of the concrete mix due to the ball-bearing effect of the fine entrained air bubbles, which reduce friction between aggregate particles [99]. As a result, the mix became easier to handle in terms of mixing, transporting, and placing. This enhancement in workability could be particularly beneficial in low-slump concrete mixes, where achieving the desired consistency can be challenging. Even a 0.04% addition increases the mix slump by 50% (from 60 mm to 90 mm). At 0.2% admixture, the slump was three times that of the *N0* mix, resulting in a change in consistency class from *S2* to *S4*. Further increases in the admixture content did not significantly affect the mix’s consistency, as the slump difference between *N2* and *N3* was only 30 mm, despite the substantial increase in the admixture dosage.

The increase in concrete mix workability, expressed as an increase in fluidity, was also reported in the literature [100,101,102].

#### 3.1.2. Compressive Strength and Ultrasonic Pulse Velocity of Concrete

**Figure 8:**
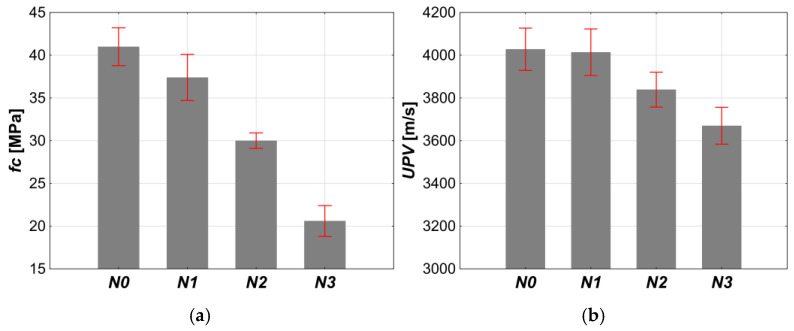
The influence of the air-entraining admixture on the: (**a**) compressive strength, (**b**) ultrasonic pulse velocity.

**Figure 9:**
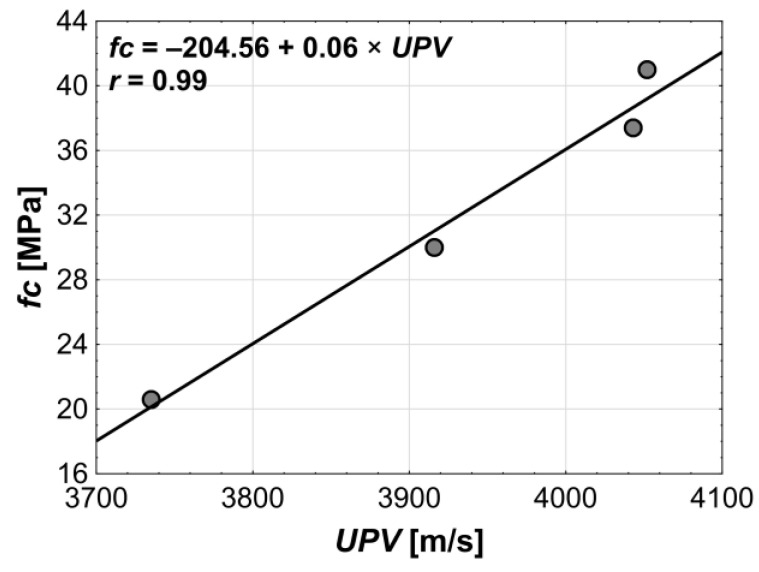
Relation between compressive strength and ultrasonic pulse velocity.

The results of compressive strength (*fc*) and ultrasonic pulse velocity (*UPV*) test after 28 days of curing are shown in Table 8 and in Figure 8 and Figure 9. The air-entraining admixture at a content of 0.04% (*N1*) caused a slight reduction in compressive strength. However, due to overlapping standard deviations, the strength of *N0* and *N1* can be considered similar, so they can be treated as part of the same data set. When the admixture content increased to 0.2% (*N2*), the compressive strength dropped significantly—by about 27% compared to *N0*. A further increase in the admixture dosage to 1.5% (*N3*) resulted in another decrease in the *f_c_*, with a total reduction of around 50%, compared to *N0*.

Changes in *UPV* with increasing admixture content were consistent with the changes in *fc*. After adding 0.04% of the admixture, the ultrasonic pulse velocity changed slightly. At 0.2% the *UPV* was reduced by about 3.0% compared to *N0*. While 1.5% caused a further decrease in the *UPV* by only about 5%, in total, the *UPV* decreased by approximately 8% compared to *N0*. Both the changes in the *fc* and the *UPV* indicated that the air introduced into the concrete mix remained in the hardened concrete as air voids. These voids were responsible for the reduction in compressive strength and the pulse velocity as the admixture dosage increased.

In general, higher porosity has a negative impact on the compressive strength, as air voids reduce the effective load-bearing area. The relation between *f_c_*-porosity in cement composites is well-documented in the literature [1,79,103,104,105].

Higher porosity is also associated with lower ultrasonic pulse velocity, making it a sensitive indicator of the density and internal structure of cement composites. As the porosity increases, more internal discontinuities appear, which disrupt the uniform transmission of ultrasonic pulses. These voids scatter and cause multiple reflections of the wavefront, increasing travel time and reducing the *UPV*. Some of the wave energy is also absorbed by the porous structure, which lowers the signal’s amplitude and clarity. This fact is also well supported in the literature [79,106,107,108].

Correlation analysis confirmed that compressive strength and pulse velocity are statistically significantly related, as the correlation coefficient was very high (*r* = 0.99). Both properties are positively correlated, meaning that an increase in one of them is accompanied by an increase in the other.

#### 3.1.3. Freeze–Thaw Resistance of Concrete

**Figure 10:**
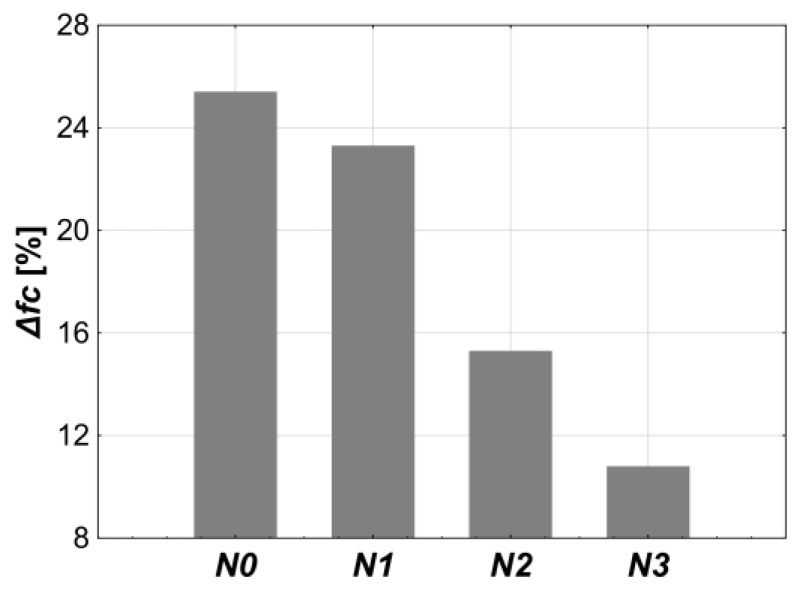
The influence of the air-entraining admixture content on the freeze–thaw resistance.

**Figure 11:**
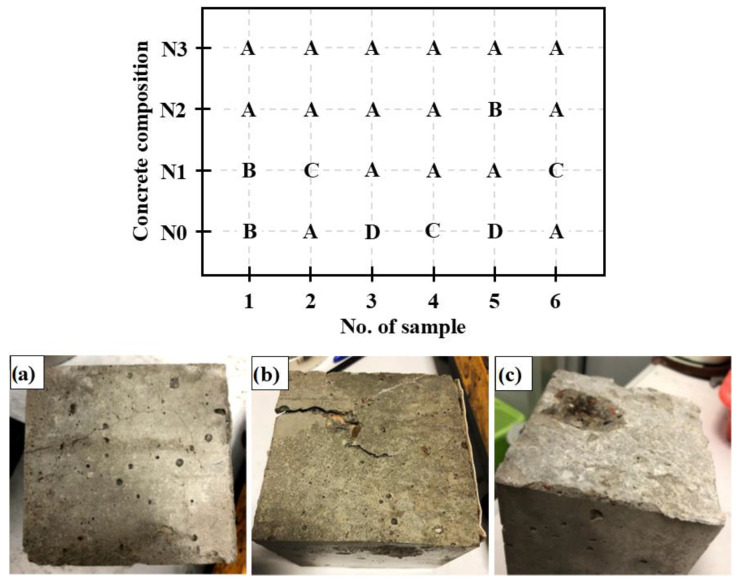
Classification of surface damages after freeze–thaw cycles. Scale of damages: A—lack of damages, B—minor cracks (**a**), C—severe cracking (**b**), and D—cracking and spalling (**c**).

The freeze–thaw resistance was evaluated based on the average mass loss (Δ*m*), the average compressive strength reduction (Δ*fc*), and the level of surface damage. The obtained results are presented in Table 9 and Figure 10 and Figure 11. The maximum average mass loss after the cycles for all concrete compositions was about 0.3%. This is well below the 5.0% limit specified by the PN-B06265 standard, which is one of the three conditions required to achieve freeze–thaw resistance.

The increase in the air-entraining admixture dosage caused changes in the average reduction in compressive strength after the cycles, indicating the improvement in the freeze–thaw resistance of the tested concrete. At a dosage of 0.04% (*N1*), the strength loss (Δ*fc* = 23.5%) was only slightly lower than in the reference concrete *N0* (Δ*fc* = 25.4%). However, with 0.2% of admixture (*N2*) the noticeable decrease in Δ*fc* occurred, bringing it below the 20% threshold necessary to confirm the freeze–thaw resistance. A further increase in the admixture dosage to 1.5% (*N3*) caused an additional decrease in Δ*fc*, reaching 10.8%.

The level of surface damage to the samples caused by cyclic freezing and thawing also shows a decreasing trend with the increasing amount of the air-entraining admixture. The highest number of damaged samples and the most severe damage were observed in the case of the reference concrete (*N0*, 4/6 damaged samples with the presence of cracks and spalling). With the admixture dosage of 0.04%, damage reduced to 3 out of 6 samples. At 0.2%, only 1 sample was slightly cracked. A further increase in the admixture content to 1.5% (*N3*) completely eliminated surface damage in the samples. The results of the visual assessment of the samples confirm the positive effect of the air-entraining admixture on freeze–thaw resistance, even at the lowest applied dosage.

Analyzing the results in light of the requirements of the PN-B06265 standard, it was found that only the *N3* concrete composition directly meets all the criteria, i.e., the average mass loss below 5%, the average compressive strength loss below 20%, and the absence of visible damage. However, *N2* may be more consistent with the definition of freeze–thaw resistance. It had only minor surface damage (the single crack on one of six samples) and still met the 20% strength loss limit after the cycles. *N2* better fits the definition of defined performance concrete, being not only durable but also structurally reliable. This is because, unlike *N3*, which lost nearly 50% of its compressive strength after the admixture was added, *N2* showed the smaller reduction of about 27%.

The positive influence of air-entraining admixture on freeze–thaw resistance has also been reported in the literature [109,110,111,112], including studies in which freeze–thaw resistance was evaluated according to PN-B-06265 [83].

#### 3.1.4. Pore Structure of Concrete

**Figure 12:**
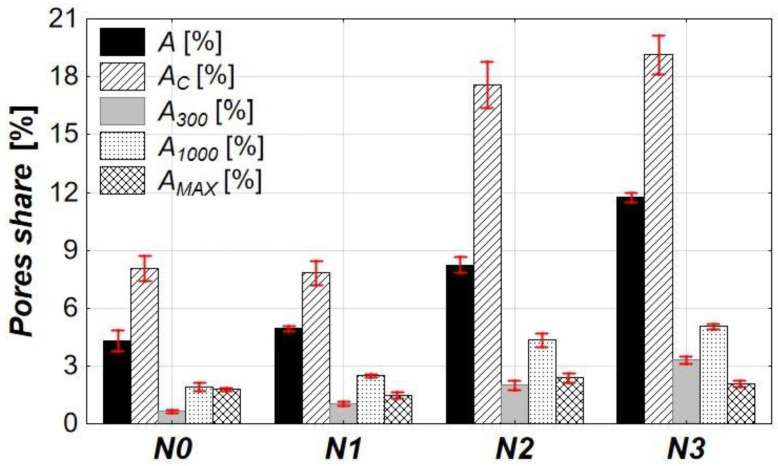
The influence of the air-entraining admixture dosage on the pore structure.

**Figure 13:**
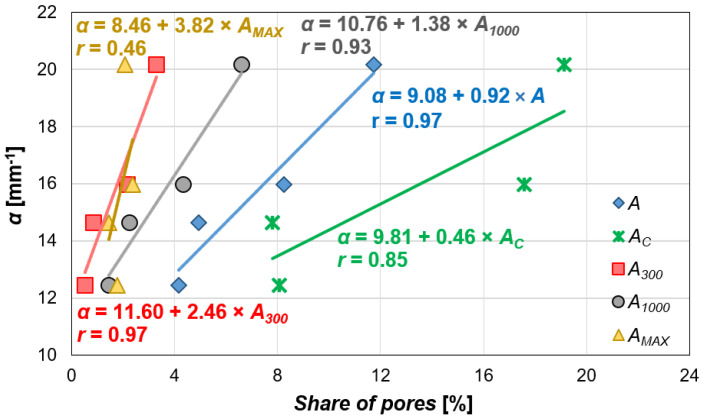
The relation between the content of pores in different size ranges and the specific surface area.

**Figure 14:**
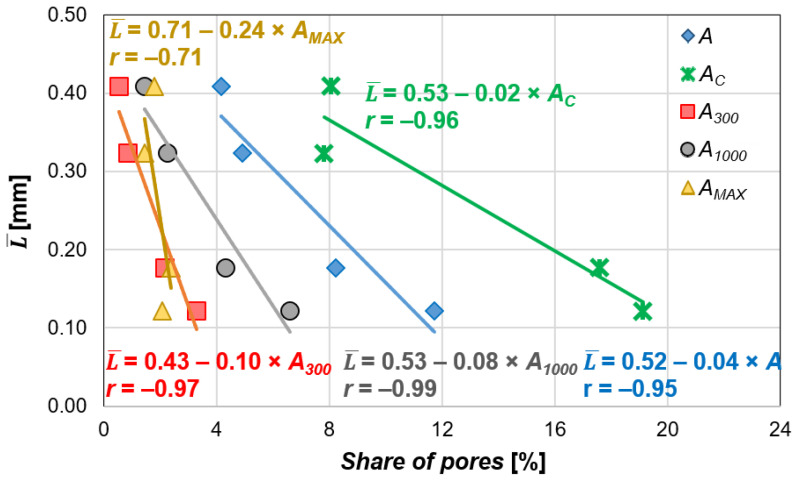
The relation between the content of pores in different size ranges and the spacing factor.

The microstructure of concrete was analyzed using the stereological method of image analysis supported by computer software. The analysis included the calculation of the total air content (*A*), the content of pores with a maximum diameter of 0.3 mm (*A_300_*), the specific surface area of pores (*α*), and the pore arrangement ratio ($\bar{L}$) which were used to assess freeze–thaw resistance according to the EN 480-11 standard. Additionally, the content of pores with the diameters ranging from 0.3 mm to 1.0 mm (*A_1000_*), those larger than 1.0 mm (*A_MAX_*), and the total porosity excluding the coarse aggregate influence (i.e., total porosity of the mortar—*A_C_*) were also determined. The results of this analysis are presented in Table 10 and Figure 12, Figure 13 and Figure 14.

The values of all computed parameters changed with an increasing dosage of the air-entraining admixture in the concrete mix. The most noticeable changes occurred at the dosage of 0.2% (*N2*). In contrast, the changes in the microstructure parameters resulting from dosing the admixture at a level five times lower (0.04%, *N1*) or over seven times higher (1.5%, *N3*) were considerably less intense. Overall, increasing the admixture content resulted in an increase in *A* in the hardened concrete, along with increases in the other related parameters (*A_C_*, *A_300_*, *A_1000_*, *A_MAX_*). A small increase in *A* was observed at the dosage of 0.04% (*N1*), while a significant increase occurred after adding 0.2% (*N2*) of the admixture. The 1.5% dosage (*N3*) caused the further increase in *A*, but it was less pronounced—in total, *A* was nearly 2.5 times higher than in the reference mix (*N0*).

The *A_C_* parameter was calculated excluding the presence of aggregate, meaning the analysis assumed no coarse aggregate was present in the concrete (no aggregate with grain sizes above 4 mm). Because of this, the *A_C_* values are much higher than *A*. However, the trend in changes remains similar. The significant increase in *A_C_* was observed only at the dosage of 0.2% (*N2*), where the *A_C_* nearly doubled compared to the *N0*, and at the 1.5% (*N3*), where it increased by almost 2.5 times.

The increase in the share of pores smaller than 0.3 mm is evident, as these are precisely the pores that should be introduced by the air-entraining admixture. The addition of 0.04%, 0.2%, and 1.5% of the admixture resulted in an increase in *A_300_* by more than 1.5, 4, and 6 times, respectively, compared to the reference sample (*N0*). The increase in *A_1000_* changed less significantly than *A_300_*. It was less pronounced—approximately 1.7 times higher at 0.04%, nearly 3 times at 0.2%, and around 3.5 times at 1.5%. This may be due to the merging of smaller pores into larger ones. In contrast, a small increase in the share of the macropores with a diameter greater than 1.0 mm after adding 0.2% (*N2*) and 1.5% (*N3*) of the admixture was rather random and most likely resulted from non-uniform compaction of the samples.

The specific surface area of pores (*α*), calculated across the entire range of air void sizes, is closely linked to the air content in hardened concrete. As new air voids are introduced, their combined surface area increases. The addition of the air-entraining admixture led to increases in α by approximately 18%, 28%, and 80% for admixture dosages of 0.04% (*N1*), 0.2% (*N2*), and 1.5% (*N3*), respectively. Correlation analysis confirmed a strong relation between the α and the total air content (*A*), with a correlation coefficient of 0.97 (Figure 13). Among the other pore-related parameters (*A_C_*, *A_300_*, *A_1000_*, and *A_MAX_*), the strongest correlation was found between the specific surface area and the share of pores with a diameter below 0.3 mm, also with a correlation coefficient of 0.97. This is due to the higher surface-to-volume ratio of smaller pores, and as a consequence, an increase in their volume share leads to a more significant increase in the total specific surface area. his is further supported by the slope coefficients of the linear regression equations, where the highest value corresponds to the α–*A_300_* relation (Figure 13). On the other hand, the weakest correlation was observed between *α* and *A_MAX_* (*r* = 0.46), which seems reasonable, as pores larger than 1 mm are typically random and result from insufficient compaction rather than admixture content.

Another parameter that changes with the increasing admixture content is the spacing factor $\bar{L}$. This factor reflects the maximum distance between any point in the cement paste and the nearest air void. As the number of pores increases, the distances between neighboring pores decrease, resulting in a lower $\bar{L}$. The addition of the air-entraining admixture reduced $\bar{L}$ by approximately 22% (*N1*), 56% (*N2*), and 71% (*N3*) compared to the reference concrete *N0*, corresponding to the admixture dosage of 0.04%, 0.2%, and 1.5%, respectively. A strong correlation was observed between $\bar{L}$ and *A* (*r* = 0.95, Figure 14). The most significant influence on $\bar{L}$ come from the pores smaller than 0.3 mm, which are introduced directly as a result of the air-entraining admixture, and from pores between 0.3 mm and 1.0 mm, which may result from either the admixture’s effect or the merging of smaller pores. This is supported by the highest slope coefficients in the linear regression equations describing the relations between $\bar{L}$ and these two parameters (*A_MAX_* was excluded due to the least reliable relation).

The analysis of quantitative parameters of microstructure (*A*, *A_300_*, *α*, $\bar{L}$) with respect to the PN-EN 480-11 standard allows for the indirect evaluation of concrete freeze–thaw resistance. According to the literature, freeze–thaw-resistant concrete typically meets the following criteria: *A* = 4–7%, *A_300_* > 1.5%, *α* = 15–24 mm^−1^, and $\bar{L}$ < 0.2–0.25 mm [14,69,70,71,72,73,74,75,76,77,78]. The results show that three out of four criteria (*A_300_*, α and $\bar{L}$) are met by the concrete *N2* and *N3*. However, none of them met the requirement regarding the total air content in hardened concrete; the closest is *N2*, which exceeded the upper limit by 1.24%. Interestingly, the *A* requirement is met by other samples, including the reference concrete *N0*, suggesting that the recommended range for this parameter might be too low for air-entrained concretes. Another possible explanation is the overestimation of *A* due to the influence of *A_MAX_*, which is highly dependent on the degree of compaction. When the *A_MAX_* is subtracted from *A*, the adjusted total air content falls within the required 4–7% range.

### 3.2. Analysis of Relations Between the Concrete Properties and the Quantative Parameters of Pore Structure

#### 3.2.1. The Influence of the Pore Structure on the Compressive Strength

**Figure 15:**
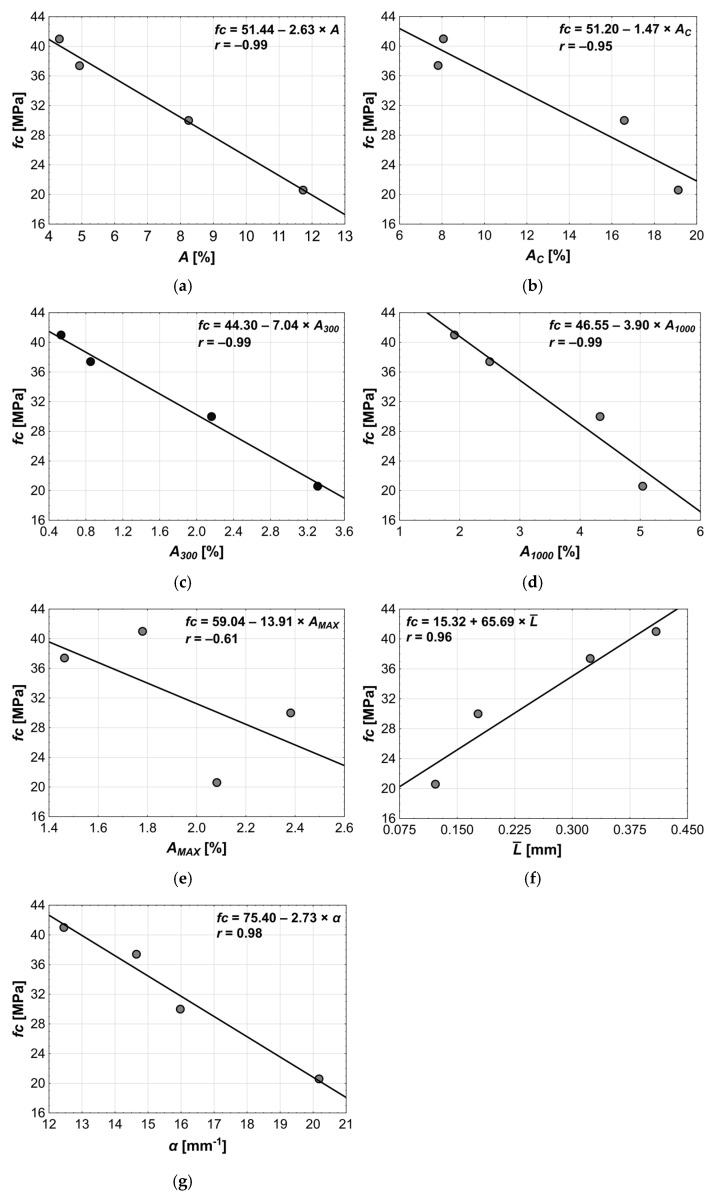
Results of linear regression analysis between *fc* and following explanatory variables: (**a**) *A*, (**b**) *A_C_*, (**c**) *A_300_*, (**d**) *A_1000_*, (**e**) *A_MAX_*, (**f**) $\bar{L}$, (**g**) *α*.

The compressive strength showed a negative correlation with all calculated microstructure parameters regarding pore content, meaning that an increase in *A*, *A_C_*, *A_300_*, *A_1000_,* or *A_MAX_* resulted in a decrease in *fc* (Figure 15). In general, all relations—except fc—A_MAX_—were characterized by correlation coefficient in the range 0.96–0.99, which classifies them as very strong relations (Figure 15a–d). The correlation of *fc* with *A_MAX_* (Figure 15e) was the weakest, as the content of pores in this category is rather incidental. However, *r* = −0.61 still indicated that these pores have some influence on compressive strength.

A very strong correlation was observed between *fc* and $\bar{L}$ (r = 0.96, Figure 15f). It was expected, as the spacing factor is closely related to the pore content in concrete. Lower pore content results in fewer pores, meaning that they are farther apart, which leads to higher compressive strength.

Another microstructural parameter strongly correlated with compressive strength is the specific surface area (*r* = 0.98, Figure 15g). An increase in *α* led to a decrease in the *fc*, as the increase in the specific surface area directly indicates a rise in the number of pores of various sizes.

The study of the influence of pore structure on compressive strength was also carried out by the multiple regression analysis with the stepwise forward selection. The following parameters were chosen as explanatory variables for the dependent variable *fc*: the total pore content (*A*), the total pore content in mortar (*A_C_*)—parameter calculated excluding the influence of coarse aggregate, the content of micropores with a diameter smaller than 0.3 mm (*A_300_*), the content of micropores with a diameter between 0.3 mm and 1.0 mm (*A_1000_*), the content of pores with a diameter greater than 1.0 mm (*A_MAX_*), the specific surface area of pores (*α*), and the spacing factor of air voids ($\bar{L}$).

The results of the successive steps of the regression analysis are shown in Table 11 and Table 12. The correlation coefficient in the first step was *R* = 0.9999 with the standard estimation error *SEE* = 0.3901 (Table 11). It turned out that the most important variable for predicting the compressive strength was the content of pores *A_1000_*, with the slope coefficient *β* = −0.9994 and the probability *p* < 0.0001. The increase in the *A_300_* caused a decrease in *fc*, similar to changes observed in the correlation analysis (see Figure 15c).

In the next step, a bivariate model was tested using the two independent variables that showed the highest correlation with *fc* (Table 12). This model included the *A_1000_* and the *A_300_* as predictors and showed the *R* = 0.9999 and the *SEE* = 0.0022. The still-high correlation coefficient and the standard estimation error two orders of magnitude lower indicated a better fit to the empirical data compared to the previous model. Moreover, the probability *p* of the *fc*(*A_1000_*, *A_300_*) model was over six times lower than the *fc*(*A_1000_*) model, confirming its higher predictive reliability.

It also is worth noting that the entire relation in the bivariate model was primarily driven by the presence of the *A_1000_* in the equation, as indicated by its standardized regression coefficient (*β* = 1.63), which was more than 2.5 times higher than that of the *A_300_*(*β* = 0.63). This suggests that pores in the 0.3–1.0 mm range have a stronger negative impact on compressive strength. The reason is that smaller pores—especially uniformly distributed—are more favorable for concrete performance, whereas an increase in pore diameter reduces the concrete’s load-bearing capacity. However, the results also showed that even the smallest pores (i.e., below 0.3 mm) can negatively affect mechanical properties, if their amount is too high. This observation is consistent with findings reported in the literature [1,2,3,4,5,6,7,8,9,10,11,12,13].

Adding any other pore structure parameter into the regression model resulted in a only a slight improvement in statistics (*R*, *SEE*) while significantly deteriorating the model’s *p*-value. Moreover, none of the additional variables were statistically significant (i.e., the variables *p*-values increased above the accepted significance level equal 0.05). These findings confirm that the bivariate regression model *f_c_*(*A_1000_*, *A_300_*) was the most optimal configuration. Due to its high quality, it can be effectively used to predict compressive strength.

#### 3.2.2. The Influence of the Pore Structure on the Ultrasonic Pulse Velocity

**Figure 16:**
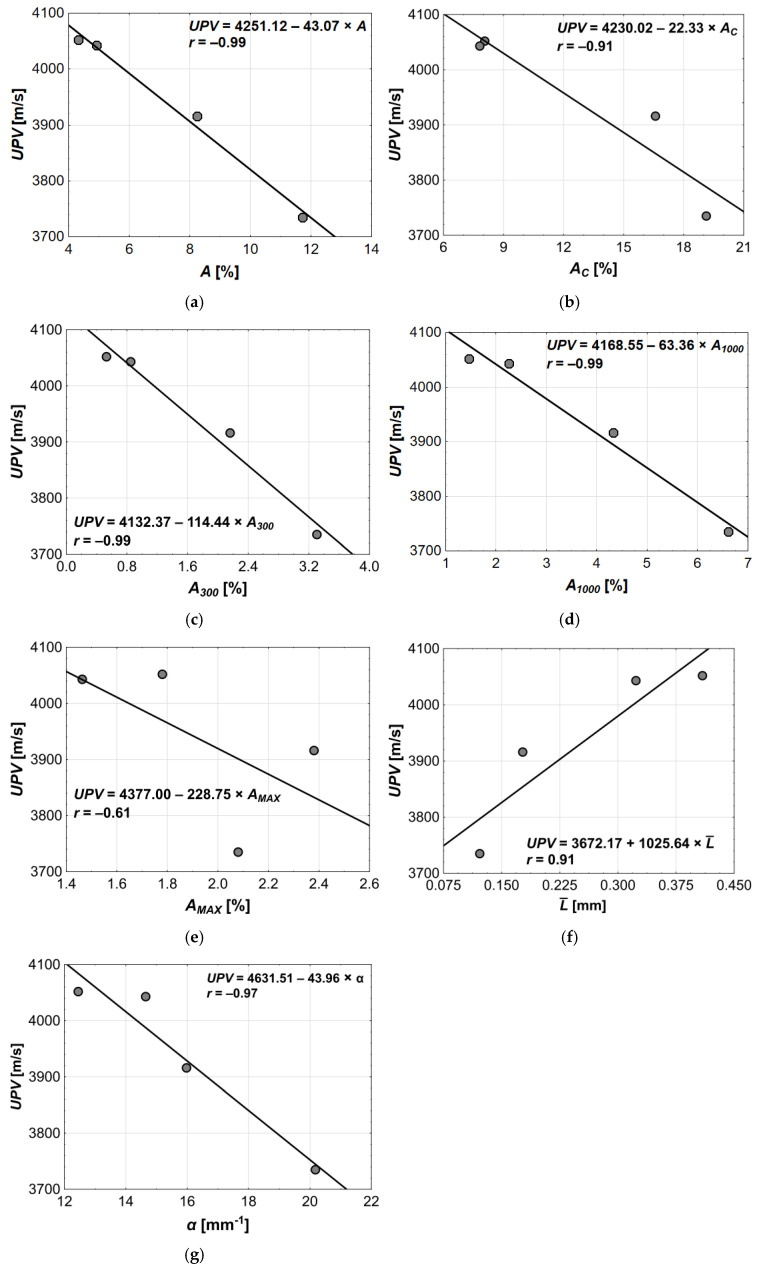
Results of linear regression analysis between *UPV* and following explanatory variables: (**a**) *A*, (**b**) *A_C_*, (**c**) *A_300_*, (**d**) *A_1000_*, (**e**) *A_MAX_*, (**f**) $\bar{L}$, (**g**) *α*.

The correlation analysis of the ultrasonic wave velocity and the pore structure parameters yielded results similar to those obtained for the compressive strength (Figure 16). The *UPV* testing plays an important role in concrete structure diagnostics, including as an indirect method for assessing compressive strength in concrete structures. This is because *fc* and the *UPV* are strongly related and, therefore, show similar correlations with other variables.

The increase in *A*, *A_C_*, *A_300_*, and *A_1000_* values led to a reduction in the *UPV* (Figure 16a–d), with correlation coefficients reaching very high values, ranging from −0.91 to −0.99. The weakest correlation was observed between the *UPV* and the macropores content *A_MAX_* (*r* = −0.61, Figure 16e). It was expected, as increasing porosity led to more internal discontinuities, which disrupt the uniform transmission of ultrasonic pulses. Air voids scatter and cause multiple reflections of the wavefront, increasing travel time and reducing the *UPV*.

The *UPV* also showed good or very good correlation with $\bar{L}$ (*r* = 0.91, Figure 16f) and the *α* (*r* = −0.97, Figure 16g). Similarly to *fc*, the increase in $\bar{L}$ and *α* resulted in an increase and decrease in the *UPV*, respectively. Both $\bar{L}$ and *α* are associated with the concrete porosity. The lower pore content corresponds to the lower number of pores, which means the average distance between them is greater and their specific surface area is smaller. That is why the decrease in $\bar{L}$ and increase in *α* led to the lower compressive strength and, consequently, the lower ultrasonic pulse velocity. Therefore, the ultrasonic method can be a good indicator of the quality of air-entrained concretes.

The influence of pore structure on the ultrasonic pulse velocity was also investigated using a multiple regression analysis with a stepwise forward selection (Table 13). The parameters selected as explanatory variables for the *UPV* were identical to those chosen for *fc*, including the total pore content (*A*), the total pore content in mortar (*A_C_*)—a parameter calculated excluding the influence of coarse aggregate—the micropore content with a diameter smaller than 0.3 mm (*A_300_*), the micropore content with a diameter between 0.3 mm and 1.0 mm (*A_1000_*), the pore content with a diameter greater than 1.0 mm (*A_MAX_*), the specific surface area of pores (*α*), and the air void spacing factor ($\bar{L}$).

The results of the successive steps of the regression analysis are presented in Table 13 and Table 14. The variable that best explains ultrasonic pulse velocity was the total pore content, as it was the first to be included in the model, which achieved the *R* = 0.9946, the *p* < 0.0054, and the *SEE* = 18.8740. The *p*-value for the variable *A* is 0.0054, indicating high statistical significance. Moreover, the negative sign of the regression coefficient (*β* = −0.9946) indicates that the increase in pore content led to a decrease in ultrasonic pulse velocity, which is consistent with the results of the correlation analysis (Figure 16a).

In the second step of the multiple regression, the model was expanded to include the *A_300_* pore content. The resulting model, *UPV* (*A*, *A_300_*), maintained the very high multiple correlation coefficient (*R* = 0.9999), the *p*-value reduced by half (*p* < 0.0027), and the standard error (*SEE* = 0.6939) two orders of magnitude lower compared to the *UPV* (*A*) model. These properties indicate that the two-variable model is better than the single-variable one.

It should also be noted that both variables in the *UPV* (*A*, *A_300_*) model were statistically significant, as their *p*-values were below the assumed significance level of 0.05 (*p* = 0.0105 for *A*, and the *p* = 0.0166 for *A_300_*). The regression coefficients indicated that changes in *A* (*β* = −2.81) have more than 1.5 times greater impact on the dependent variable *UPV* compared to *A_300_* (*β* = 1.73). Among all the parameters, the total pore content *A* has the greatest influence on the *UPV*, indicating that for ultrasonic wave propagation, the overall content of air voids in concrete is more important than their size. The wave velocity depends on the material’s elasticity, which in turn is affected by the content of air voids in the concrete. The higher the porosity, the lower the elastic modulus [113], and consequently, the lower the *UPV* [114].

Furthermore, the direction of the *UPV* changes caused by both explanatory variables was opposite: the increase in A resulted in a decrease in the *UPV*, whereas the increase in *A_300_* led to the increase in the *UPV*. Notably, the influence of *A_300_* is opposite to that observed in the correlation analysis (Figure 16c).

The compressive strength and the ultrasonic pulse velocity were highly correlated, which was reflected in the similarities between their multiple regression results. In both cases, the optimal model was a two-variable regression model, in which different variables (*A_1000_* and *A_300_*) were initially included. However, in the second step, the same pore content *A_300_* was incorporated into both models.

The *UPV* (*A*, *A_300_*) model proved optimal because adding any other variable describing the pore structure, although it slightly improved the model’s properties (*R*, *SEE*), resulted in variables that were not statistically significant (*p* > 0.05), and similarly, the model’s *p*-value worsened. The performance of the developed model demonstrates its high quality, suggesting that it can be effectively used for predicting ultrasonic pulse velocity.

#### 3.2.3. The Influence of the Pore Structure on the Freeze–Thaw Resistance

**Figure 17:**
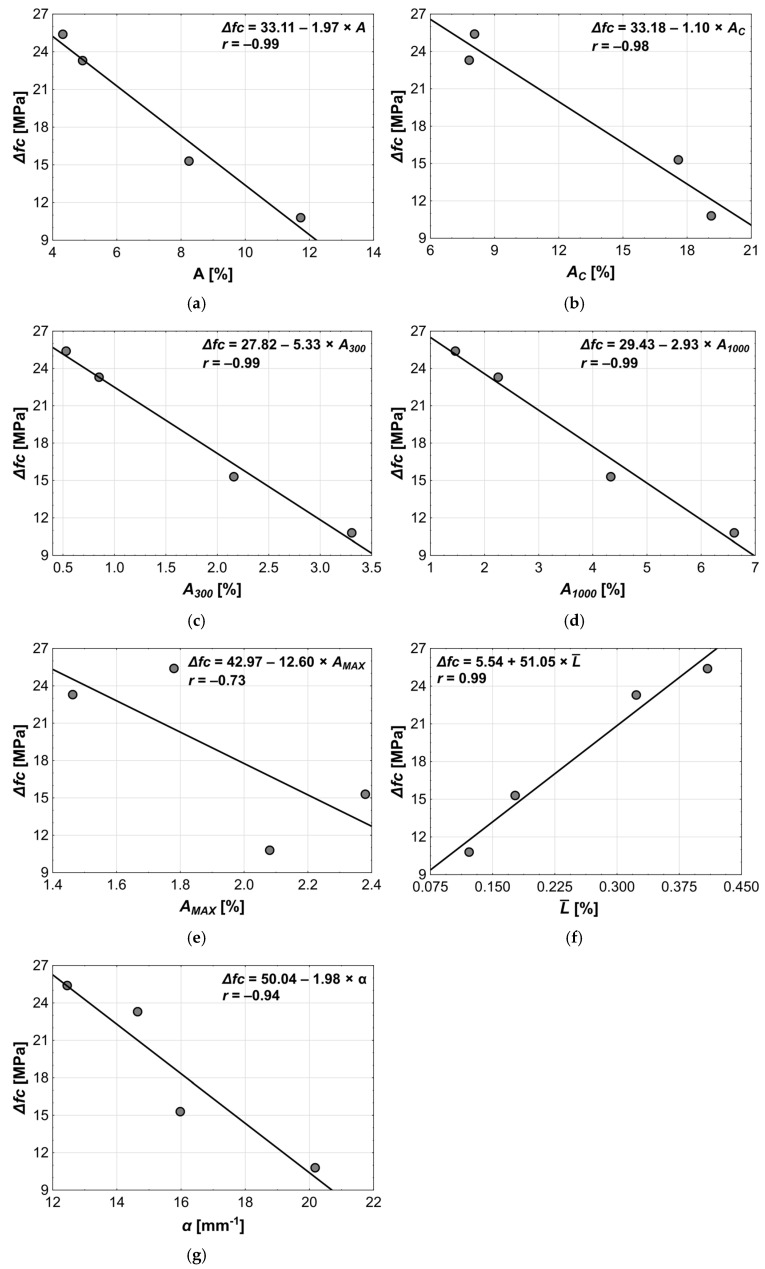
Results of linear regression analysis between Δ*fc* and following explanatory variables: (**a**) *A*, (**b**) *A_C_*, (**c**) *A_300_*, (**d**) *A_1000_*, (**e**) *A_MAX_*, (**f**) $\bar{L}$, (**g**) *α*.

The freeze–thaw resistance of concrete, expressed as the percentage loss in compressive strength between reference samples and samples subjected to the freeze–thaw cycles, showed very strong correlation with all the microstructure parameters (*r* ranging 0.98–0.99, Figure 17a–d,f,g), except for the *A_MAX_ (*Figure 17e), which exhibited a significantly lower correlation coefficient (*r* = –0.87). The increase in the values of parameters *A*, *Ac*, *A_300_*, and *A_1000_* resulted in a decrease in the Δ*fc*, with the strongest effect observed for the *A_300_*—the highest slope coefficient (5.33)—more than twice that of the *A_1000_* (2.93). The decrease in the Δ*fc* indicated the increase in freeze–thaw resistance, as the freeze–thaw cycles caused a progressively smaller reduction in the compressive strength. The dominant influence of the *A_300_* parameter on the Δ*fc* confirmed the well-known benefit of introducing air bubbles up to 0.3 mm in diameter in cement composites. Since increasing the amount of air-entraining admixture raised both the *A_300_* and the *A_1000_* pore contents, the Δ*fc* showed similarly strong correlations with all parameters describing porosity.

The compressive strength change (Δ*fc*) was also very strongly correlated with the air void spacing factor $\bar{L}$ (*r* = 0.99) and the specific surface area of pores *α* (*r* = −0.99). The increase in Δ*fc*—that is, a decrease in resistance to freeze–thaw cycles—was observed alongside the decrease in the $\bar{L}$ and the increase in *α*. This, in turn, is related to the increase in pore content—in other words, the increase in the number of pores, which reduced the distance between them and increased their total specific surface area. The strong correlation of both parameters with Δ*fc* underscored the usefulness of determining their values when assessing the freeze–thaw resistance of concrete.

It should also be noted that each of the pore structure parameters identified in the literature as significant from the standpoint of freeze–thaw resistance (*A*, *A_300_*, $\bar{L}$, *α*) was similarly strongly associated with Δ*fc*.

The analysis of the influence of pore structure on the freeze–thaw resistance was also conducted using multiple regression analysis with stepwise forward selection. The parameters selected as explanatory variables for Δ*fc* were identical to those chosen for *fc* and *UPV*, including the total pore content (*A*), the total pore content in mortar (*A_C_*), the micropore content with a diameter smaller than 0.3 mm (*A_300_*), the micropore content with a diameter between 0.3 mm and 1.0 mm (*A_1000_*), the pore content with a diameter greater than 1.0 mm (*A_MAX_*), the specific surface area of pores (*α*), and the air void spacing factor ($\bar{L}$).

The results of the regression analysis for the freeze–thaw resistance are presented in Table 15. The microstructure parameter that best explained changes in the Δ*fc* is the share of pores with a diameter up to 0.3 mm (*p* = 0.0008). This parameter is the only variable that appeared in the regression model, because no other parameter added as the first or second one was statistically significant. Just like in the correlation analysis, the regression analysis also showed that the increase in *A_300_* resulted in the decrease in Δ*fc*, which was related to the increase in freeze–thaw resistance. The Δ*fc* (*A_300_*) model had a very good fit to the data, indicated by very good model properties—an *R* value of 0.9945, a probability *p* < 0.0055, and an *SEE* of 0.8779.

These results showed that among the pore structure parameters listed in EN 480-11, *A_300_* best describes the freeze–thaw resistance evaluated according to the PN-B-06250 standard. This may suggest that *A_300_* alone might be enough for an indirect assessment of freeze–thaw resistance. Moreover, the developed model is of sufficient quality to be used for estimating the level of freeze–thaw resistance.

## 4. Conclusions

The goal of this study was to examine the influence of concrete modification with the air-entraining admixture on its properties and pore structure. It also analyzed the relations between the pore structure, described by quantitative stereological parameters, and selected properties, namely compressive strength, ultrasonic wave velocity, and freeze–thaw resistance. To achieve this, correlation analysis and multiple regression analysis with forward stepwise selection were used. Based on the obtained results, the following conclusions can be drawn:

– The image analysis method, supported by custom software, was successfully used to describe the air void structure in concrete.
– The effectiveness of the air-entraining admixture was confirmed. It increased the air content in the fresh concrete mix and improved its workability. The entrained air remained in the hardened concrete as pores—mostly smaller than 1.0 mm. This led to the decrease in the compressive strength and the ultrasonic pulse velocity, but also improved the concrete’s resistance to freeze–thaw cycles.
– The optimal admixture dosage was found to be 0.2% of the cement mass. At this level, the concrete showed the best balance between compressive strength and freeze–thaw resistance, confirmed by both indirect (pore structure) and direct (strength loss after freezing–thawing cycles) methods.
– The ultrasonic pulse velocity proved to be a useful tool for evaluating the air entrainment quality, as it strongly correlates with both compressive strength and pore structure parameters defined in EN 480-11.
– The pore structure parameter that best explains changes in compressive strength is the content of pores between 0.3 and 1.0 mm. The smaller pores—especially those uniformly distributed—are more favorable for concrete performance, whereas an increase in pore diameter reduces concrete’s load-bearing capacity.
– The total pore content is the best predictor of ultrasonic pulse velocity, indicating that for ultrasonic wave propagation, the overall content of air voids in concrete is more important than their size. The wave velocity depends on the material’s elasticity, which in turn is affected by the content of air voids in the concrete. The higher the porosity, the lower the elastic modulus, and consequently, the lower the *UPV*.
– For the freeze–thaw resistance (measured directly), the most important pore structure parameter is the content of pores smaller than 0.3 mm. This indicates that pores of this size are crucial for maintaining concrete durability under cyclic freezing and thawing conditions. It also confirms the effectiveness of the air-entraining admixture, which introduces air voids into the concrete—specifically those smaller than 0.3 mm.
– The high quality of the regression models developed using stepwise forward selection showed that they can be used to predict compressive strength, ultrasonic pulse velocity, and freeze–thaw resistance based on stereological pore structure analysis.
– The approach used in this study could be the first step toward fully automating the evaluation of freeze–thaw resistance by the image analysis method. The next step may involve applying deep learning algorithms.

## Figures and Tables

**Table 1:** Characteristics of air-entraining admixture [91].

Characteristic	Content [%]
Form	Liquid
Color	Straw yellow
Density	1.003 ± 0.01 kg/dm^3^
pH	7 ± 1
Chloride ion content	≤0.1%
Alkali content	≤1.0%

**Table 2:** The composition of concretes tested in the study.

Symbol	Ingredients			
	Cement [kg]	Water [kg]	Aggregate [kg]	Air-Entering Admixture [% of Cement Mass]
*N0*	320	177	1905	0.00
*N1*				0.04
*N2*				0.20
*N3*				1.50

**Table 3:** Consistency classes according to slump test method [92].

Class	Slump Flow [mm]
S1	10–40
S2	50–90
S3	100–150
S4	160–210
S5	≥220

**Table 4:** The fundamental parameters used to describe objects in a material’s cross-section and throughout its volume.

Parameter	Definition
*A_A_*	Surface area of objects related to an image area
*L_A_*	Line length related to an image area
*N_A_*	Number of objects related to an image area
*V_V_*	Volume fraction of the selected objects in a volume unit of a material
*S_V_*	Surface area of selected objects in a volume unit of a material
*L_V_*	Line length in a volume unit of a material
*N_V_*	Number of elected objects in a volume of a material

**Table 5:** Summary of parameters obtained for tested concretes by image analysis method.

Parameter	Short Description
*A*	Volume fraction of pores across full range of pore sizes
*A_300_*	Volume fraction of pores of sizes below 0.3 mm
α	Specific surface area of pores across full range of pore sizes
$\bar{L}$	Average maximum distance from any point in cement paste to edge of nearest pore
*A_1000_*	Volume fraction of pores of sizes between 0.3 mm and 1.0 mm
*A_MAX_*	Volume fraction of pores of sizes above 1.0 mm
*A_C_*	Volume fraction of pores across full range of pore sizes, excluding volume of coarse aggregate

**Table 6:** Summary of conducted tests and samples used for each composition.

Test	Sample Size (mm^3^)	No. of Samples
Freeze–thaw resistance	100 × 100 × 100	12
Compressive strength	100 × 100 × 100	3
Ultrasonic pulse velocity	100 × 100 × 100	3
Pore structure, i.e., air contents, specific surface area, spacing factor	75 × 75 × 10	3

**Table 7:** Air content (*AC*) and consistency (*ST*) of concrete mixes.

Sample	*AEA* [%]	*AC* [%]	*ST* [mm]
*N0*	0.00	2.6	60
*N1*	0.04	2.9	90
*N2*	0.20	5.9	180
*N3*	1.50	9.4	210

*AEA*—Air-entraining admixture content [%].

**Table 8:** Compressive strength (*fc*) and ultrasonic pulse velocity (*UPV*) of concretes.

Sample	*AEA* [%]	*fc* [MPa]	*UPV* [m/s]
*N0*	0.00	41.0 ± 2.2	4052 ± 48
*N1*	0.04	37.4 ± 2.7	4043 ± 59
*N2*	0.20	30.0 ± 0.9	3916 ± 86
*N3*	1.50	20.6 ± 1.8	3735 ± 76

*AEA*—Air-entraining admixture content [%].

**Table 9:** The air content and consistency of concrete mixes.

Sample	*AEA* [%]	Δ*m* [%]	Δ*fc* [%]
*N0*	0.00	0.2	25.4
*N1*	0.04	0.1	23.5
*N2*	0.20	0.1	15.3
*N3*	1.50	0.3	10.8

*AEA*—Air-entraining admixture content [%].

**Table 10:** Results of quantitative analysis of microstructure described by stereological parameters.

Sample	*AEA* [%]	*A* [%]	*A_C_* [%]	*A_300_* [%]	*A_1000_* [%]	*A_MAX_* [%]	α [mm^−1^]	$\bar{L}$ [mm]
*N0*	0.00	4.32 ± 0.54	8.06 ± 0.67	0.53 ± 0.10	1.46 ± 0.22	1.78 ± 0.09	12.45 ± 0.53	0.41 ± 0.02
*N1*	0.04	4.93 ± 0.14	7.81 ± 0.63	0.85 ± 0.12	2.50 ± 0.08	1.46 ± 0.16	14.65 ± 0.98	0.32 ± 0.05
*N2*	0.20	8.24 ± 0.39	16.59 ± 1.21	2.16 ± 0.25	4.33 ± 0.34	2.38 ± 0.18	15.97 ± 0.47	0.18 ± 0.02
*N3*	1.50	11.73 ± 0.24	19.13 ± 1.01	3.31 ± 0.21	5.04 ± 0.15	2.08 ± 0.24	22.45 ± 0.97	0.12 ± 0.01

*AEA*—Air-entraining admixture content [%].

**Table 11:** Results of multiple regression analysis for variable *A_1000_* (step 1).

*N* = 4	Dependent variable—*fc* *R* = 0.9994; *p* < 0.0006; *SEE* = 0.3901 *fc* = 46.55 − 1.00 × *A_1000_*				
	*β*	*SD β*	*B*	*SD B*	*p*
*A_1000_*	−0.9994	0.0249	−3.9041	0.0976	<0.0001
Constant			46.5492	0.4072	0.0006

**Table 12:** Results of multiple regression analysis for variable *A_1000_* and *A_300_* (step 2).

*N* = 4	Dependent variable—*fc* *R* = 0.9999; *p* < 0.0001; *SEE* = 0.0022 *fc* = 47.93 − 6.37 × *A_1000_ +* 4.47 × *A_300_*				
	*β*	*SD β*	*B*	*SD B*	*p*
*A_1000_*	−1.6304	0.0026	−6.3691	0.0100	<0.0001
*A_300_*	0.6319	0.0026	4.4664	0.0181	0.0026
Constant			47.9330	0.0061	<0.0001

**Table 13:** Results of multiple regression analysis for variable *A* (step 1).

*N* = 4	Dependent variable—*UPV* *R* = 0.9946; *p* < 0.0054; *SEE* = 18.8740 *UPV* = 25.13 − 3.19 × *A*				
	*β*	*SD β*	*B*	*SD B*	*p*
*A*	−0.9946	*A*	−0.9946	*A*	−0.9946
Constant		Constant		Constant	

**Table 14:** Results of multiple regression analysis for variable *A* and *A_300_* (step 2).

*N* = 4	Dependent variable—*UPV* *R* = 0.9999; *p* < 0.0027; *SEE* = 0.6939 *UPV* = 4453.98 − 117.89 × *A +* 200.75 *× A_300_*				
	*β*	*SD β*	*B*	*SD B*	*p*
*A*	−2.7220	*A*	−2.7220	*A*	−2.7220
*A_300_*	1.7305	*A_300_*	1.7305	*A_300_*	1.7305
Constant		Constant		Constant	

**Table 15:** Results of multiple regression analysis for variable *A_300_* (step 1).

*N* = 4	Dependent variable—Δ*fc* *R* = 0.9945; *p* < 0.0055; *SEE* = 0.8779 Δ*fc* = 27.81 − 5.33 × *A_300_*				
	*β*	*SD β*	*B*	*SD B*	*p*
*A_300_*	−0.9945	*A_300_*	−0.9945	*A_300_*	−0.9945
Constant			27.8149	0.8094	0.0008

## Data Availability

The original contributions presented in this study are included in the article. Further inquiries can be directed to the corresponding author.
